# Renal and Red Marrow Dosimetry in Peptide Receptor Radionuclide Therapy: 20 Years of History and Ahead

**DOI:** 10.3390/ijms22158326

**Published:** 2021-08-03

**Authors:** Stephan Walrand, François Jamar

**Affiliations:** Cliniques Universitaires Saint-Luc, 1200 Brussels, Belgium; francois.jamar@uclouvain.be

**Keywords:** somatostatin, PRRT, dosimetry, DOTATOC, DOTATATE

## Abstract

The development of dosimetry and studies in peptide receptor radionuclide therapy (PRRT) over the past two decades are reviewed. Differences in kidney and bone marrow toxicity reported between ^90^Y, ^177^Lu and external beam radiotherapy (EBRT) are discussed with regard to the physical properties of these beta emitter radionuclides. The impact of these properties on the response to small and large tumors is also considered. Capacities of the imaging modalities to assess the dosimetry to target tissues are evaluated. Studies published in the past two years that confirm a red marrow uptake in ^177^Lu-DOTATATE therapy, as already observed 20 years ago in ^86^Y-DOTATOC PET studies, are analyzed in light of the recent developments in the transferrin transport mechanism. The review enlightens the importance (i) of using state-of-the-art imaging modalities, (ii) of individualizing the activity to be injected with regard to the huge tissue uptake variability observed between patients, (iii) of challenging the currently used but inappropriate blood-based red marrow dosimetry and (iv) of considering individual tandem therapy. Last, a smart individually optimized tandem therapy taking benefit of the bi-orthogonal toxicity-response pattern of ^177^Lu-DOTATATE and of ^90^Y-DOTATOC is proposed.

## 1. Introduction

Peptide receptor radionuclide therapy (PRRT) is a well-established therapy for metastatic cancers expressing somatostatin receptors. A somatostatin analog peptide, such as octreotide or octreotate, is covalently bounded to a chelator, such as DTPA or DOTA. This chelator can be viewed as an empty basket. Before intravenous injection, a radionuclide (^111^In, ^90^Y, ^177^Lu) is mixed with the chelator–peptide in a solution at a low pH and at a temperature both optimized to promote the entrance of the radionuclide within the chelator cage. High labelling fractions are easily reached, i.e., less than 2% of residual free radionuclide. The labeled compound exhibits a rather high stability when the pH solution is increased above 6.

The quantity to be injected is expressed in becquerel (Bq), which corresponds to one decay per second, and is named activity. By analogy with pharmaceutical drug, this quantity is often named dose, which is incorrect—the term dose identifies the quantity of energy deposed in a tissue by the particles emitted during the decays. This dose is expressed in gray (Gy), corresponding to 1 joule (J) delivered in 1 kg of tissue.

The first PRRT trials used ^111^In-DTPA-octreotide [1], a tracer initially developed for diagnostic intent. Due to the cell internalization of the tracer, the short range ^111^In auger electrons were considered suitable for tumor therapy [2]. The observed tumor control appeared promising, but an escalating activity study quickly revealed hematological concerns [3]. Indeed, as most of the ^111^In decay energy is converted into gamma rays, the cross-irradiation of the red marrow from the remainder of the body was significant [4].

This issue was overcome using ^90^Y-DOTA-octreotide (^90^Y-DOTATOC) [5], which for therapy intent, can be considered as a pure beta emitter. This was confirmed by the ^90^Y-DOTATOC clinical phase I trial SMT-487 [6,7]. With this compound, the first organ at risk was no longer the red marrow, but the kidneys, which is less life threatening [8]. However, due to its long beta range, i.e., maximal 11 mm in water, the dose delivered to small tumors is limited. Thus, relapse of micro-metastases initially not visible on imaging modalities were observed, although all known metastases completely responded.

To improve small tumor control, ^177^Lu-DOTA-octreotate (^177^Lu-DOTATATE) was developed [9], the beta of which has a smaller range, i.e., 2 mm in water. Although the decay energy is mostly brought by the beta, the main tissue at risk identified in the phase III study was the red marrow, with about 10% of the patients undergoing grade 3 or 4 lymphopenia [10].

The aim of this paper is to provide a comprehensive review of the literature evidencing and explaining the different toxicity and response patterns observed between ^90^Y-DOTATOC and ^177^Lu-DOTATATE PRRT. Last, by combining therapy improvements reported in the literature, a smart optimized tandem therapy design is proposed.

## 2. Kidney Dosimetry and Toxicity

The ^90^Y-DOTATOC clinical phase I trial ^86^Y-SMT-487 [6,7,8] was designed following the FDA requirements. The total injected activity was computed to deliver a maximum dose of 27 Gy to the kidneys, based on a pre-therapy ^86^Y-DOTATOC PET using the MIRD pamphlet no. 11 [11], which assumed a standard kidney volume for the dosimetry calculation. The PET scan was reconstructed using a dedicated prompt single gamma ray correction [12].

**Figure 1:**
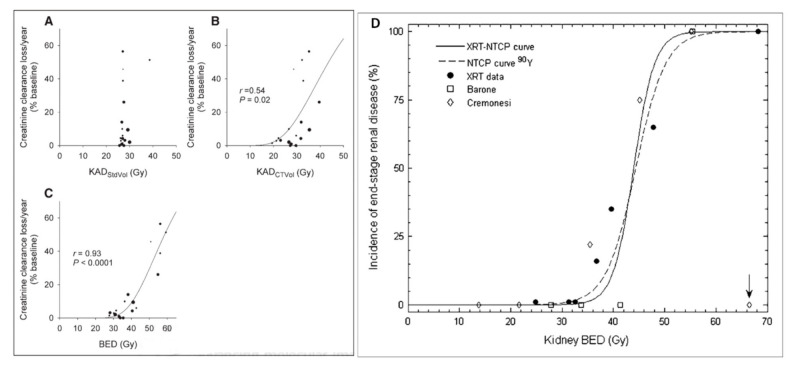
(**A**) Toxicity observed in the ^86^Y-SMT-487 phase I trial as a function of the absorbed dose computed with the MIRD pamphlet no. 11 formalism [11], (**B**) after rescaling with the individual kidney volume and (**C**) converted into BED. The dots diameter corresponds to the number of cycles. Reprinted with permission from Ref. [8]. Copyright 2021 SNM. (**D**) Matching of the NTCPs observed in ^90^Y-DOTATOC with that of EBRT. Reprinted with permission from Ref. [13]. Copyright 2021 SNM.

The trial included a cycle activity escalation, i.e., a reduction in the number of therapy cycles needed to reach 27 Gy to the kidneys, starting from five cycles to a single cycle. The patient kidney follow-up was set as 5 years, with patients exhibiting a creatinine clearance lost per year up to 60%. Figure 1A clearly shows that obviously no toxicity–dose correlation was observed, as all the patients, apart from one receiving an extra cycle for compassionate use, received the same kidney dose.

Rather than to conclude that dosimetry was useless, which sometimes happens in nuclear medicine, the patient CT analogic films were scanned and the renal volume measured. At this time, no hybrid SPECT-CT or PET-CT was available, and due to limited data storage capacity, most patient CT slices were only analogically archived. Figure 1B shows that by just rescaling the kidney dose with the standard to individual kidney volume ratio, a toxicity–dose relation became visible. 

It was noted that globally, the patients receiving fewer cycles experienced higher creatinine clearance lost per year (Figure 1B). The absorbed doses were thus converted into a biological effective dose (BED) using the Lea–Catcheside formalism [14], and a clear toxicity–dose correlation appeared (Figure 1C). This toxicity–dose relation observed in nuclear medicine was the first one matching that observed in EBRT (Figure 1D): the dose calculation based on fractionation commonly used in EBRT was proved applicable in nuclear medicine.

In all PRRT studies, i.e., ^111^In-DTPA-octreotide SPECT [15], ^86^Y-DOTATOC PET [12] and ^177^Lu-DOTATATE SPECT [16], showed that the kidney uptake is localized in the cortex such as initially observed by Hammond et al. in planar ^111^In-DTPA-octreotide scintigraphy [17]. Intra-patient ^86^Y-DOTATOC PET studies proved that amino acid infusion significantly reduced the renal reuptake [6]. By competing with the megalin/cubilin complex on the apical membrane of proximal tubular cells (PTCs), basic amino acids, such as L-lysine and L-arginine, can reduce by ≈50% the reuptake of the radiolabeled peptide. The other fraction is taken up by the PTCs by fluid-phase endocytosis, that is not influenced by the presence of high amounts of basic amino acids [18,19]. Studies in knock-out rat provided evidence that megalin is essential for renal tubule reabsorption of the peptide [20]. 

**Figure 2:**
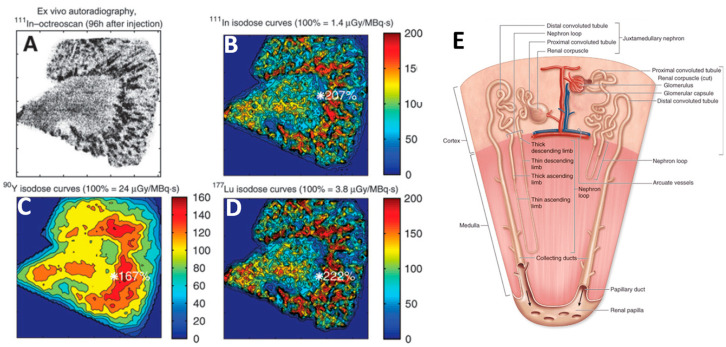
(**A**) ^111^In autoradiography. (**B**–**D**) corresponding isodoses simulated by Monte Carlo for ^111^In, ^90^Y and ^177^Lu, respectively. Reprinted with permission [21] 2021 SNM. (**E**) nephron anatomy showing the glomerulus and tubule location. Reprinted from [22].

Ex vivo autoradiography of healthy kidney tissue, from patients who received ^111^In-DTPA-octreotide before nephrectomy (Figure 2A), showed an uptake gradient decreasing from the inner to the outer cortex boundary [21]. As the radiosensitive glomerulus is about 6 mm far from the inner boundary (Figure 2E), its crossfire irradiation by the activity taken up by the tubules will strongly depend on the radionuclide beta range, as clearly shown in the Monte Carlo (MC) isodose simulations (Figure 2B,C). These MC simulations explain why the first limiting tissue is the kidney with ^90^Y-DOTATOC and the red marrow in ^177^Lu-DOTATATE PRRT, respectively (see Section 3).

## 3. Red Marrow Dosimetry and Toxicity

A highly variable red marrow inter-patient uptake in PRRT was evidenced early in 2005 during the ^86^Y-DOTATOC phase I ^86^Y-SMT-487 trial, which used state-of-the-art PET imaging [23]. Furthermore, this red marrow uptake was intra-patient, correlated to that measured with ^111^In-DTPA-octreotide by SPECT [24]. This was overlooked by the nuclear medicine community, who argued that RM uptake was not visible in ^90^Y bremsstrahlung or ^177^Lu SPECT [25,26]. The case appeared to be solved in 2017 when the LutatheraTM insert package, which states that establishing red marrow dosimetry is useless in predicting observed toxicity, was agreed upon by the FDA and the EMA [27].

These past few years, several teams using state-of-the-art SPECT/CT demonstrated clear red marrow uptake of ^177^Lu in ^177^Lu-DOTATATE therapy [16], the dosimetry based on enabling some toxicity prediction [28,29,30]. The observed red marrow dosimetry was about fourfold that of the blood-based method used in the Netter study [10], explaining why about 10% Grade 3–4 hematological toxicity was observed in this study.

Such red marrow uptake could appear surprising, given the high DOTA affinity and stability for yttrium and for lutetium. However, in the transchelation competition with transferrin, DOTA is just a passive and naïve chelator stroke by an active and cunning thief. Iron is a vital compound for mammalians and evolution spent hundreds of millions of years to improve transferrin, versus a few decades for chemists.

**Figure 3:**
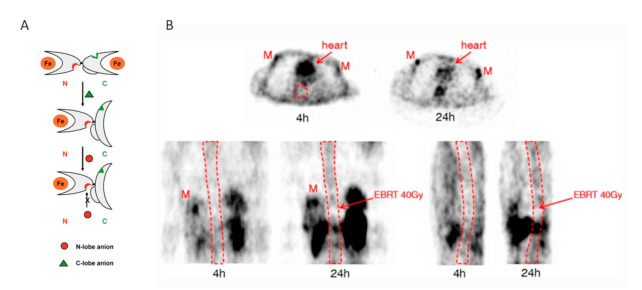
(**A**) Transferrin release mechanism (Reprinted from Ref. [31]) (**B**) 24 h post ^86^Y-DOTATOC injection from the ^86^Y-SMT-487 clinical trial. Metastases (M) already exhibit a strong uptake 4 h pi, while red marrow activity behaves in phase opposition to the blood pool visible in the heart. Previously irradiated vertebra by EBRT, which has eradicated active marrow, have a similar behavior as the blood pool. Reprinted with permission from Ref. [24]. Copyright 2021 Springer.

Transferrin is a protein having two active lobes (Figure 3A) [31]. In the iron-free state, i.e., apo-transferrin, the N-lobe and the C-lobe are open, ready to catch a metallic ion. When a metallic ion enters a lobe, the lobe closes and surrounds the ion as a result of the Van der Waals forces. In this state, no other external force can easily remove the ion from the locked lobe. When an appropriate anion binds to the corresponding active site, the lobe opens and releases the metallic ion. This key-padlock mechanism ensures that iron will be released to the appropriate cells and not elsewhere in the body.

The ion transferrin release is an active process, along with the ion catching one. Inorganic chelators, such as DOTA, have to wait for the release of a metal ion by another chelator in order to trap it, which is rare from a good competitor. In contrary, kinetics and spectrometry studies evidenced that transferrin can create a ternary (or mixed) complex with the initial metal–chelator complex [32]. During the life of this ternary complex, the metal ion is transferred from the chelator to the transferrin metal binding site, likely by the Van der Waals forces. Many in vitro competition studies between transferrin and DOTA evidenced the transferrin superiority [24,33]. In vitro data using plasma from subjects injected with ^111^In-pentetreotide demonstrated that after 7 days, the radioactive metal was effectively bound to transferrin [34].

A recent study in humans [35] showed that only 23% and 2% of ^177^Lu-DOTATATE remain intact after 24 h and 96 h post-injection, respectively. Figure 3B clearly illustrates the uptake difference between the tumor receptor-based mechanism and the transferrin transport to red marrow mechanism observed in the ^86^Y-SMT-487 study [24]. Directly during the first pass, the ^86^Y-DOTATATE binds to the tumor receptors, resulting in a strong uptake within 4 h pi, while it takes a much longer time for the transferrin to catch the metal and to deliver it to the red marrow cells. The 4 and 24 h images proved the red marrow activity behavior in the opposition phase versus the blood pool, which makes the blood-based red marrow dosimetry method unlikely to be physiologically adequate. Table 1 shows red marrow dosimetry assessments and dose–toxicity correlations reported in the recent literature. 

Using 4 SPECT/CT performed after the first and second cycles, Santoro et al. [28] evaluated the organs at risk in 12 patients treated with ^177^Lu-DOTATATE. The mean dosimetry for kidney and red marrow was 0.43 ± 0.13 mGy/MBq and 0.04 ± 0.02 mGy/MBq, respectively. As the maximal tolerated dose for red marrow is about tenfold lower than that of kidney [24], this explains why hematological toxicity is the limiting factor in 26% of individually optimized ^177^Lu PRRT [36]. 

**Figure 4:**
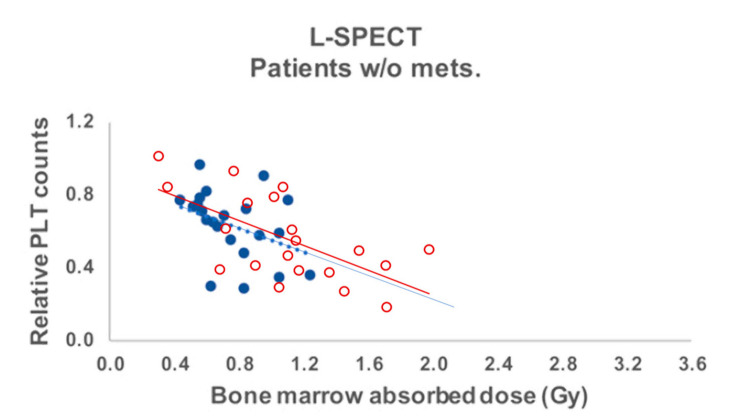
Blue solid circles: relative platelet counts decrease at nadir as a function of the red marrow in **^177^**Lu-DOTATATE therapy (Reprinted with permission from Ref. [29]. Copyright 2019 P Bernhardt). Red empty circles: decrease observed in the ^90^Y-DOTATOC trial using ^86^Y-DOTATOC PET-based dosimetry added by the authors [24]. Both trendlines are very similar.

Using a SPECT/CT-planar hybrid method, Hagmarker et al. [29] in ^177^Lu-DOTATATE therapy found similar red marrow dosimetry, i.e., 0.06 (0.02–0.12) mGy/MBq in 22 patients without skeletal metastases. They observed a dose–platelet counts nadir relationship (Figure 4) perfectly in line with that observed in the ^90^Y-DOTATOC clinical phase I trial using ^86^Y-DOTATOC PET-based red marrow dosimetry [23,24]. 

In 200 patients treated with ^177^Lu-DOTATATE, Garsk et al. [36] observed fourfold lower mean blood-based red marrow dosimetry than that observed in the two reported SPECT studies, i.e., 0.016 mGy/MBq, which was unable to predict the hematological toxicity observed in 40 patients. Page et al. [37] compared blood-based and SPECT/CT-based red marrow dosimetry in 11 patients treated with ^177^Lu-DOTATATE and observed the same fourfold ratio.

The results of these six studies [28,29,30,35,36,37] urge for using post-cycles SPECT/CT-based red marrow dosimetry for all ^177^Lu therapies. With optimized imaging and reconstruction methods of SPECT/CT, these studies provide evidence that direct RM uptake-based dosimetry clearly outperforms the blood-based estimations, as advocated in the Luthatera package insert. One must keep in mind that the maximal red marrow tolerable dose is about tenfold lower than that of other tissues. Thus, a long duration, i.e., >30 min, dual-head SPECT is required to accurately assess the red marrow uptake at several timepoints. Red marrow activity should be measured on the thoracic vertebras: the region with the lowest attenuation and without surrounding tissues with high uptake, such as the liver, spleen or kidneys, that can produce long tail artefacts in reconstructed slices. Fortunately, nowadays, a single SPECT/CT scan encompass the thorax and the kidneys. Clearly, state-of-the-art imaging capacities as available now can help improve this paradigm of RM dosimetry based on imaging, rather than on blood activity.

## 4. ^177^Lu-^90^Y PRRT Tandem Therapy: A Bi-Orthogonal Synergy

Hobbs et al. [38] introduced the “orthogonal radionuclides” concept to promote tandem therapy with radionuclides taken up by different tissues and thus presenting different toxicities. Such tandem allows increasing the tumor dose by splitting the unwanted irradiation between different organs, as similarly performed in EBRT by rotating the beam source around the targeted tumor. 

Comparing toxicities of ^177^Lu-DOTATATE and ^90^Y-DOTATOC therapies is very challenging considering the sparse information provided in the clinical trials publications. In the Netter phase III study [10], in 116 patients receiving four repeated injections of 7.4 GBq every 8 weeks, 0.4% and 11% underwent a renal and hematological toxicity of grade 3 or 4, respectively. In the phase II ^90^Y-DOTATOC study [39] in 1109 patients receiving in average 2.5 cycles of 3.7 GBq/m^2^, 9.2% and 12.8% experienced a renal and hematological toxicity of grade 3 or 4, respectively. However, the frequency of this hematological toxicity cannot be directly compared; indeed, assuming similar uptakes, the ^90^Y protocol delivered a much higher tissue dose than that of ^177^Lu (Table 2). The hematological to renal toxicity ratio is about twentyfold higher for the ^177^Lu-DOTATATE therapy.

Regarding dosimetry-based studies, in individualized ^177^Lu-DOTATATE study aiming to deliver 23 Gy to the kidney in 154 patients, 26% presented a hematological toxicity, halting the therapy [36], while this event occurs only in 1 patient out of 60 in the individualized ^90^Y-DOTATOC study aiming to deliver 27 Gy to the kidney [7]. Again, a twentyfold higher prevalence of hematological toxicity is observed in ^177^Lu-DOTATATE therapy.

This much higher prevalence of hematological toxicity in ^177^Lu-DOTATATE therapy is in line with the low in vivo stability of this compound: only 23% and 2% of ^177^Lu-DOTATATE remain intact after 24 h and 96 h post-injection in patients [35], whereas 90% of ^86^Y-DOTATOC remains intact 5 h post-injection in primates [40]. Intuitively, it makes sense that the larger electron orbital of ^177^Lu makes it more sensitive to external Van der Waals forces coming from an open transferrin lobe. Note that, as transferrin is not excreted by the kidneys, radionuclide to protein binding analysis in urine is not appropriate to evaluate the in vivo compound stability. In vitro transchelation competition with apo-transferrin could also be biased, considering that in vivo, apo-transferrin is continuously renewed by the release of the metal ion in the red marrow. Such in vitro studies thus have to be performed with a huge apo-transferrin overload. Last, caution is also needed with in vivo rodent stability studies, considering that experiments proved different iron binding and release properties compared to humans [41]; this could also hold true for yttrium and lutetium.

All these facts clearly show that the orthogonal toxicity concept applies to the couple ^177^Lu-DOTATATE and ^90^Y-DOTATOC for which the prime tissue at risk is the red marrow and the kidney, respectively. This feature alone should be sufficient to promote the tandem approach as a new standard for PRRT therapy.

Last, but not least, this tandem is also orthogonal regarding the tumor response (justifying the bi-orthogonal appellation): by its short beta range, ^177^Lu is efficient to deliver high doses in sub-centimetric tumors, whereas the ^90^Y beta, with its one cm range, is more efficient to cross-irradiate low vascularized regions often present in larger tumors [42,43].

**Figure 5:**
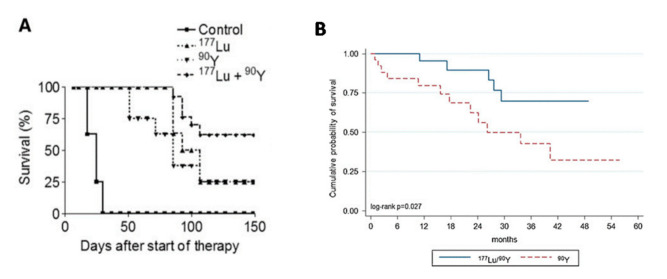
(**A**) Survival in rats bearing a small and large tumor treated with single or tandem PRRT. Reprinted with permission from Ref. [44]. Copyright 2021 SNM. (**B**) Survival in patients treated with 7.4 GBq/m^2^ of ^90^Y-DOTATOC or with 3.7 GBq/m^2^ of ^90^Y-DOTATOC + 3.7 GBq/m^2^ of ^177^Lu-DOTATATE. Note that assuming similar uptake, the tandem delivers absorbed doses 30% lower than those of ^90^Y alone. Reprinted with permission from Ref. [45]. Copyright 2021 Springer.

This dose–response synergy was already observed in 2005 in a preclinical model [44]. Pancreatic cancer cells were successively grafted, i.e., spaced by 3 weeks, in the two opposite flanks of rats, resulting in rats bearing a small (≈0.5 cm^2^) and a large (≈8 cm^2^) tumor. Rats were split into four groups of about 12 individuals. Survival was impressively higher in rats treated with the ^177^Lu-DOTATATE and ^90^Y-DOTATOC tandem than that of rats treated with only one radionuclide (Figure 5A).

Kunikowska et al. [45] compared overall survival (OS) between single ^90^Y-DOTATOC therapy and ^90^Y-^177^Lu tandem therapy. The patients were not randomly drawn: the first 25 consecutive patients were treated with ^90^Y alone (7.4 GBq/m^2^), and the following 25 consecutive patients were treated with the ^90^Y-^177^Lu tandem (3.7–3.7 GBq/m^2^). However, the same patient enrollment criteria were chosen for both groups. As in the rat study, the OS was impressively better in the tandem group, especially that, when assuming similar uptake, the tandem delivers absorbed doses 30% lower than that of the ^90^Y alone using this activity protocol.

In this study, the tandem was given together in two or three cycles, which is not the right strategy: the well-vascularized large tumor region takes fewer radionuclides in the following cycle due to the additional ^177^Lu irradiation eradicating cells which uptake the peptide. The best strategy is to first perform the ^90^Y cycles to benefit from the high uptake of vascularized regions to cross-irradiate the other tumor regions, then to end up with a ^177^Lu cycle to efficiently irradiate, and hopefully eradicate, small metastases [46]. 

## 5. Individual PRTT Optimization Planning

Multi-cycle PRRT is well adapted to individualized therapy planning as the first cycle delivered dose is safe for all patients. Afterwards, post-cycle dosimetry can be performed to optimize the activity to be injected in the following cycles. Note that if ^98^Ga-DOTATATE PET is of prime interest to select a candidate for the PRRT therapy, it is useless for dosimetry estimation given its too short half-life. In ^90^Y-DOTATOC therapy, phantom and patient studies proved ^90^Y PET imaging providing an accurate kidney dose estimation [47], while medium energy collimator SPECT/CT is well adapted for ^177^Lu-DOTATATE [16], provided that the acquisition time is long enough.

PRRT dosimetry requires at least two imaging time points, as tissue activity curves exhibit a bi-exponential asymmetric bell shape. This feature renders proposed single time-point estimates inaccurate [48], which is only for a single exponential starting from t = 0, the model used to propose it [49]. One time point after the maximal uptake is needed, e.g., 24 h post-injection, and another time point somewhere around one effective half-life, i.e., 48 or 72 h post ^90^Y injection and 5 to 8 days post ^177^Lu injection.

Only the two tissues at risk have to be imaged, i.e., the thoracic vertebrae and the kidneys, which can be performed with two PET/CT positions for ^90^Y and with one single SPECT/CT position for ^177^Lu. Planar imaging and combined planar and hybrid SPECT/CT should be avoided given the superimposition of the residual blood-pool activity for red marrow and of the liver and of the spleen activity for the kidneys. Many physicians think it is mandatory to use state-of-the-art TOF-PET/CT to perform tumor staging or follow-up! It is time to tackle claims that short-duration, whole-body planar scan is sufficient to perform dosimetry. By always trying to simplify dosimetry to satisfy this claim, medical physics experts (MPE) are left with poor quality data for accurate dosimetry, which reinforces the common belief that dosimetry is useless.

Menda et al. [50] conducted a prospective post-cycle renal dosimetry using ^90^Y-bremsstrahlung SPECT/CT in 25 patients with neuroendocrine tumors. A ^90^Y TOF-PET/CT [47] was used at the first time for bremsstrahlung SPECT/CT calibration purposes. The study confirmed the very high variability of inter-patient renal dosimetry, as already observed using ^86^Y-DOTATOC PET [8], advocating the interest to individually optimize the injected ^90^Y activity.

Almost twenty years after the individualized ^90^Y-SMT-487 trial [23], Del Petre et al. [51], Sundlov et al. [52] and Garske-Roman et al. [36] performed SPECT/CT-based individualized planning in ^177^Lu-DOTATATE therapy in 52, 51 and 200 patients aiming at a renal BED = 27 Gy, D = 23 Gy and D = 23 Gy, respectively. They demonstrated that applying the standard recommendation of four 7.4 GBq cycles results in undertreating 85%, 73% and 49% of the patients. 

**Figure 6:**
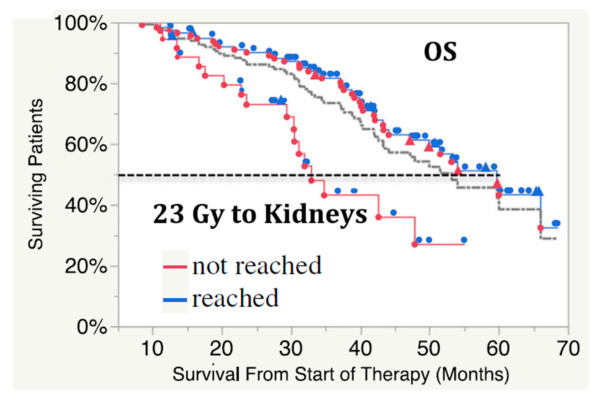
Overall survival in ^177^Lu-DOTA-octreotate therapy in relation to 23 Gy achieved to the kidney (blue curve), or not due to hematological toxicity (red curve). Gray curve: all patients OS. Red symbol: patients died. Blue symbol: patients alive (Reprinted with permission from Ref. [36]. Copyright 2021 Sundin).

Garske-Roman et al. [36] observed an impressive progression-free survival (PFS) and overall survival (OS) improvement of patients who were able to reach the 23 Gy renal dose limit (*n* = 114) versus those who could not, due to hematological toxicities (*n* = 40) (Figure 6). 

## 6. Smart Optimized Tandem Therapy Design: A Proposal

**Figure 7:**
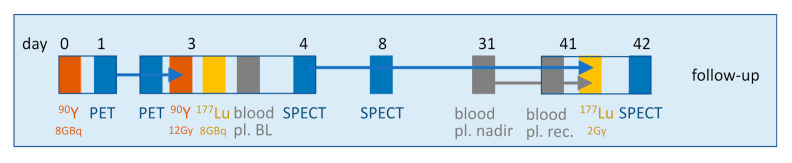
Proposed smart optimized tandem therapy work flow including 4 patient visits to the hospital, with one or two imagings at each visit.

As in tandem therapy, the potential toxicity is split between two different tissues, and the activity fractionation can be reduced versus a single radionuclide therapy, which has the benefit to reduce aggressive tumors regrowth between cycles. As a result, a smart full optimized tandem therapy, fractionated in three cycles, can be proposed (Figure 7). It requires only four patient visits to the hospital, three amino acid infusions and five imaging sessions.

At day 0, a fixed activity of ^90^Y-DOTATOC/TATE is injected with amino acid infusion. The following day, before releasing the patient from the hospital, a ^90^Y kidney 30 min-PET/CT is performed. 

At day 3, a ^90^Y kidneys 30 min-PET/CT is performed to compute the kidney dosimetry in order to assess the residual ^90^Y activity needed to reach a BED of 15 Gy to the kidneys. Afterwards, this ^90^Y-DOTATOC/TATE activity complement is injected to the patient together with a fixed ^177^Lu-DOTATATE activity along with amino acid infusion, and a blood sample is withdrawn to obtain the cell count base line. As this session corresponds to the last ^90^Y cycle, co-injection of ^177^Lu does not impact the ^90^Y cross irradiation. Note that with regard to the effective kidney half-life (≈30 h, [8]), the initial dose rate is already reduced by a factor ≈5, corresponding to an effective dose fractionation. The following day, before releasing the patient, a ^177^Lu thorax-abdomen 30 min dual-head SPECT/CT is performed. Additionally, for a highly valuable scientific point of view, a blood sample could be taken to assess the relative binding of ^177^Lu and ^90^Y to transferrin in the same patient at the same time using size-exclusion chromatography, with appropriate MW standards.

At day 8, a ^177^Lu thorax-abdomen SPECT/CT is performed, and the kidney and RM dosimetry is computed. The kidney dosimetry should be computed using the Sfactors taking into account the ^90^Y and ^177^Lu beta range for the cross irradiation of the glomerulus by the taking up tubules [21]. At day 31, a blood sample is taken by the treating team or by the general practitioner to estimate the blood cell count nadir.

At day 41, a blood sample is withdrawn to check the cell count recovery, and depending on the values at nadir and recovery, a ^177^Lu-DOTATATE activity complement is injected, satisfying, for the whole therapy, the two limits: D < 2 Gy to the red marrow and BED < 31 Gy to the kidneys. Note that the physician has the competence to modulate these limits according to the patient status and to his cell count recovery. The following day, before releasing the patient, a thorax-abdomen ^177^Lu SPECT/CT is performed.

Afterwards, the patient undergoes the normal follow-up.

## 7. Conclusions

In recent years, many studies provided evidence of the huge benefit for the patient outcome to individually optimize the activity to be injected. Furthermore, although not performed along an optimized planning, recent studies have also established the importance to profit of the bi-orthogonal toxicity-response patterns of the tandem ^90^Y-DOTATOC-^177^Lu-DOTATATE. There is no doubt that the combination of these two approaches, as proposed in Figure 7, will still improve the patient outcome and will push PRRT to a real curative intent.

However, last year, the EANM published a position paper [53] on article 56 of the Council Directive 2013/95 Euratom, which required an individualized optimization planning in all radiotherapies. This position paper aims to provide guidance on how to interpret the Directive and states that ^177^Lu-DOTATATE, used according to the package insert, is a standard therapy not requiring any individualized planning, which is scientifically questionable considering the present review. 

However, let us recall that legally, the MPE responsibility is framed by the corresponding national transposition of the directive. However, the MPE or practitioner cannot be prosecuted for having followed Directive 2013/95 rather than the national implementation. Indeed, as the Directive 2013/95 provisions are unconditional and sufficiently clear and precise, the directive has direct effects, and any individual can invoke its provisions in front of any national court [54]. The European Court of Justice’s jurisprudence has extended this principle to cases with incorrect implementation of a directive [55].

## Figures and Tables

**Table 1:** Blood and image-based RM dose comparisons.

^177^Lu-DOTATATE Study	Blood-Based RM Dosi. (mGy/MBq)	Image-Based RM Dosi. (mGy/MBq)	Toxicity–Dose Correlation
Santoro et al. [28]	n.a.	0.04 [0.01–0.09]	Yes
Hagmarker et al. [29]	n.a.	0.06 [0.02–0.12]	Yes
Garsk et al. [36]	0.016	n.a.	No
Page et al. [37]	0.02 [0.01–0.03]	0.06 [0.03–0.11]	Yes for imaging

n.a. = not assessed.

**Table 2:** Comparison of grade 3–4 toxicities between ^177^Lu-DOTATATE and ^90^Y-DODTATOC therapies.

	^177^Lu-DOTATATE Phase III [10]	^90^Y-DOTATOC Phase II [39]	^177^Lu-DOTATATE Indiv. Optim. [36]	^90^Y-DOTATOC Indiv. Optim. [7]
inj. activity	4 × 7.4 GBq	2.5 × 10.4 GBq/m^2^	23 Gy to kidneys	27 Gy to kidneys
inj. decay energy	511 J	2193 * J	n.a.	n.a.
renal toxicity	0.4%	9.2%	n.a.	n.a.
hematotoxicity	11.0%	12.8%	**26.0%**	**1.6%**
hemato/renal tox	**27.5**	**1.4**	n.a.	n.a.

*: Assuming a body surface area of 1.7 m^2^; n.a.: not applicable; indiv. optim.: individualized optimization. Bold numbers show the hematotoxicity risk comparison.

## Data Availability

All data came from published articles.
